# Thermoinduced structural-transformation and thermochromic luminescence in organic manganese chloride crystals†
†Electronic supplementary information (ESI) available: Crystallographic details, tables of crystal and refinement data for **1a** and **1b**; PXRD, TGA UV-vis diffuse reflectance spectra, photoluminescence emission spectra and photoluminescence lifetime spectra of compounds. CCDC 1867428 and 1867436. For ESI and crystallographic data in CIF or other electronic format see DOI: 10.1039/c8sc04711a


**DOI:** 10.1039/c8sc04711a

**Published:** 2019-02-25

**Authors:** Meng-En Sun, Yao Li, Xi-Yan Dong, Shuang-Quan Zang

**Affiliations:** a College of Chemistry and Molecular Engineering , Zhengzhou University , Zhengzhou 450001 , China . Email: zangsqzg@zzu.edu.cn; b College of Chemistry and Chemical Engineering , Henan Polytechnic University , Jiaozuo 454000 , China

## Abstract

The [Mn_2_Cl_9_]^5–^ mode of red emissive (C_4_NOH_10_)_5_Mn_2_Cl_9_·C_2_H_5_OH under thermal treatment will be cleaved into [MnCl_4_]^2–^ in the green emissive (C_4_NOH_10_)_2_MnCl_4_ with the departure of ethanol. The rapid conversion of luminescence from red to green provides new insight into the luminescence origin and thermal stability of organic–inorganic metal halide hybrids.

## Abstract 2

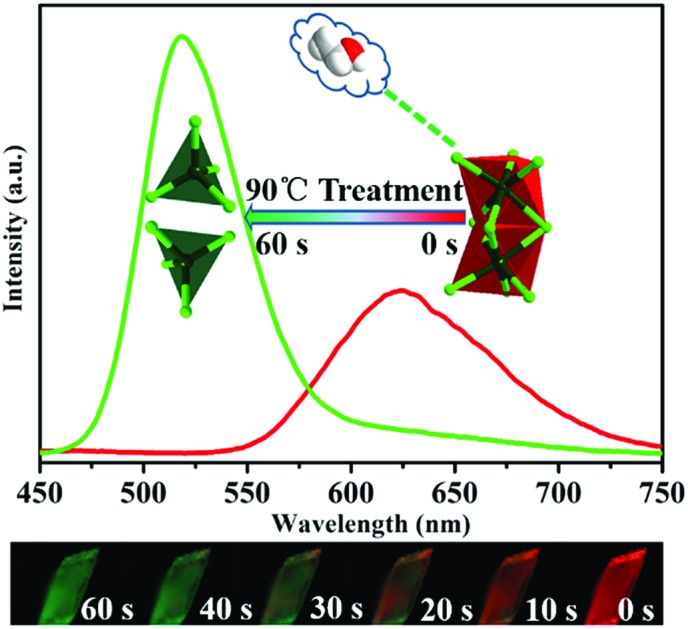
The [Mn_2_Cl_9_]^5–^ mode in the binuclear red emissive (C_4_NOH_10_)_5_Mn_2_Cl_9_·C_2_H_5_OH is cleaved into [MnCl_4_]^2–^ in the green emissive (C_4_NOH_10_)_2_MnCl_4_.

## 

Metal (Mn,^1^ Mo,^2^ W,^3^ Pb,^4^ Sn,^5^ and Cu^6^) halides (Cl, Br, and I) represent an attractive class of molecular building blocks in luminescent functional materials, which demonstrate potential application in light-emitting diodes, luminescent solar concentrators, photovoltaic modules, *etc.*^7^ Among them, organic manganese(ii) halide hybrids frequently display strong luminescence originating from the d–d transition, with lifetimes from microseconds to milliseconds.^1^,^8^ The reported manganese halides focus on monomanganese species,1a,^9^ manganese chain structure1b,^10^ and layer structure,^11^ however the oligomeric cluster of manganese(ii) remains quite limited,^12^ which deserved careful investigation due to the structural uniqueness.

Crystalline state structure transformation concomitant with a huge change of luminescence involved in chemical bond cleavage or reconstruction upon an external stimulus is very interesting, and is commonly found in framework materials,^13^ yet remains a challenge in cluster compounds.^14^ The emission in manganese(ii) halides is sensitive to their ligand-fields.^1^,^8^ To the best of our knowledge, the combination of stimuli-responsive crystalline structure transformation and luminescence conversion of oligomeric manganese halide clusters has not been reported.

Here, we assembled a binuclear red-emissive manganese chloride cluster crystal (C_4_NOH_10_)_5_Mn_2_Cl_9_·C_2_H_5_OH (**1**) using morpholine as the organic counter cation. Upon high-temperature induction, **1** crystals was fast structurally transformed into green-emissive (C_4_NOH_10_)_2_MnCl_4_ (**2**) crystals* in situ*. The crystal structural analysis revealed that the manganese dimer in octahedral coordination decomposed into mono manganese species in tetrahedral mode, accompanied by the departure of guest ethanol molecules.

Compound **1** was prepared by mixing manganese chloride (MnCl_2_) and morpholine hydrochloride (C_4_NOH_10_Cl) (2 : 5 molar ratio) in ethanol at room temperature (R.T.) (Scheme 1). The proportion of MnCl_2_ and C_4_NOH_10_Cl at 1 : 2 and the reaction temperature at 90 °C were modulated to obtain compound **2**. The precise structures of compounds **1** and **2** were determined by single-crystal X-ray diffraction (SCXRD) (Fig. 1a and b, ESI Tables S1–S4†). As depicted in Fig. 1a, the Mn^2+^ cations occupy the octahedral centre and six chloride ions are located at the vertex. The [Mn_2_Cl_9_]^5–^ dimers are face-shared through three coordination chloride ions and embedded periodically in the matrix of organic cations C_4_NOH_10_^+^. And C_4_NOH_10_^+^ counter ions and ethanol molecules are hydrogen-bonded with [Mn_2_Cl_9_]^5–^ dimers in compound **1** (ESI Fig. S1†). For compound **2**, the Mn^2+^ cations occupy the tetrahedral centre coordinated by four chloride ions (Fig. 1b). The purity of compounds **1** and **2** was confirmed by the well-overlapped powder X-ray diffraction (PXRD) patterns between the as-synthesized samples and those simulated from single-crystal data (ESI Fig. S2 and S3†).

**Scheme 1:**
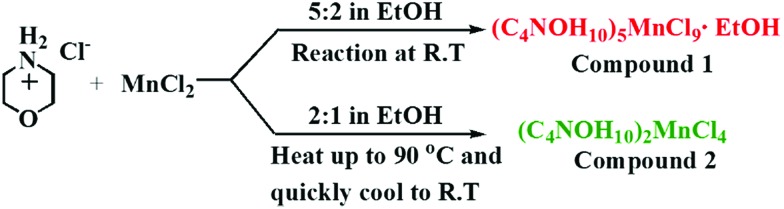
The synthesis routes to compounds **1** and **2**.

**Fig. 1:**
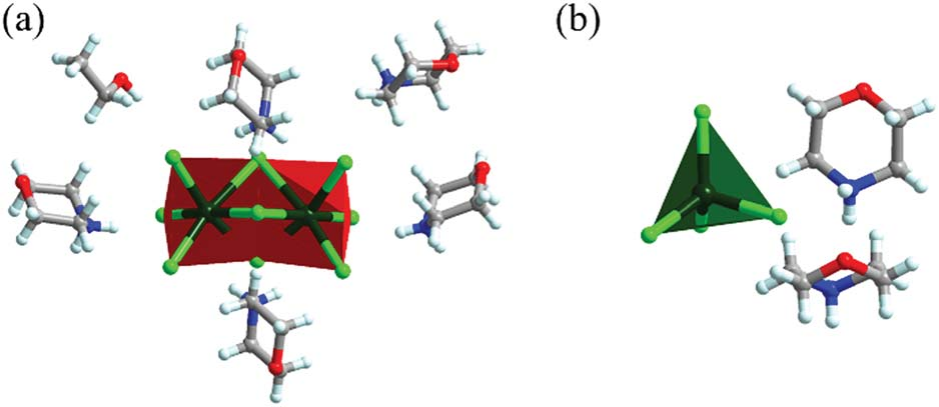
Crystal structures of compounds **1** (a) and **2** (b). Colour codes: dark green = manganese; bright green = chlorine; red = oxygen; gray = carbon; blue = nitrogen; light turquoise = hydrogen.

The photophysical properties of compounds **1** and **2** are fully characterized by UV-vis diffuse reflectance spectra and steady state and time resolved photoluminescence (PL) spectra. The peaks around 240–345 nm for **1** and **2** originate from the transitions within the C_4_NOH_10_^+^ cation, including the n–σ* and σ–σ* in the UV-vis diffuse reflectance spectra (ESI Fig. S4†). And the other peaks around 345–600 nm for **1** and **2** can be ascribed to electronic transitions between the ground and the excited states of the Mn^2+^ ion in the crystal field.^15^ The excitation peak at 520 nm nearest to the emission peak at 620 nm corresponds to a characteristic transition from the ground state of the d-electron configuration (t_2g_)^3^(e_g_)^2^ to the upper state of the configuration (t_2g_)^4^(e_g_)^1^ (ESI Fig. S5†). The excitation peak at 450 nm nearest to the emission peak at 520 nm corresponds to a characteristic transition from the ground state of the d-electron configuration (e_g_)^2^ (t_2g_)^3^ to the upper state of the configuration (e_g_)^3^ (t_2g_)^2^ (ESI Fig. S6†).^16^ The large energy separation between the emission wavelength maximum (compound **1** at 620 nm, compound **2** at 520 nm) and the nearest excitation wavelength maximum (compound **1** at 520 nm, compound **2** at 450 nm) is probably due to the forbidden transition from the ground state to the first excited triplet level. The R.T. PL quantum efficiencies of compounds **1** and **2** are 29% and 39% respectively, and characteristic PL lifetimes are 4.83 ms and 3.31 ms respectively (ESI Fig. S7 and S8†). The longer lifetime of Mn_2_ dimers might be related to the fact that the ligand field was rigidified by these hydrogen-bonding interactions (Fig. S1†), which efficiently decreased some nonradiative loss decay induced by vibrational relaxation. The emission peaks and the emission decay time of compounds **1** and **2** hardly changed at different excitation wavelengths (ESI Fig. S9, S10 and Table S5†). They further showed that the emission of compounds **1** and **2** is d–d transition (from the lowest excited triplet state ^4^T_1_ to the ground state ^6^A_1_) phosphorescent emission of the manganese(ii) ion in the d^5^ configuration.

Interestingly, compound **1** changed the PL colour from red to green upon continuous one-minute thermal treatment at 90 °C (Fig. 2a–c). The thermochromic luminescence process was recorded by fluorescence microscopy (Fig. 2a). The corresponding emission spectra exhibited the gradual disappearance of the 620 nm peak, and the simultaneous appearance of the 520 nm peak (Fig. 2b). In the Commission Internationale de l’Eclairage (CIE) coordinates (Fig. 2c), the luminescence colour of **1** gradually changed from red (0.61, 0.36) to green (0.24, 0.64) with thermal treatment time. As the luminescence colour of compound **1** after thermal treatment at 90 °C is consistent with that of compound **2** at R.T. (ESI Fig. S11†), we hypothesized that there was a thermally induced structural transformation from **1** to **2** through a reaction shown in Fig. 3a, in which compound **1** could be decomposed into compound **2**, C_4_NOH_10_Cl and ethanol. That is to say, the structure of [Mn_2_Cl_9_]^5–^ dimers in compound **1** might decompose into two units [MnCl_4_]^2–^ with the departure of ethanol molecules, producing compound **2** (Fig. 3b).

**Fig. 2:**
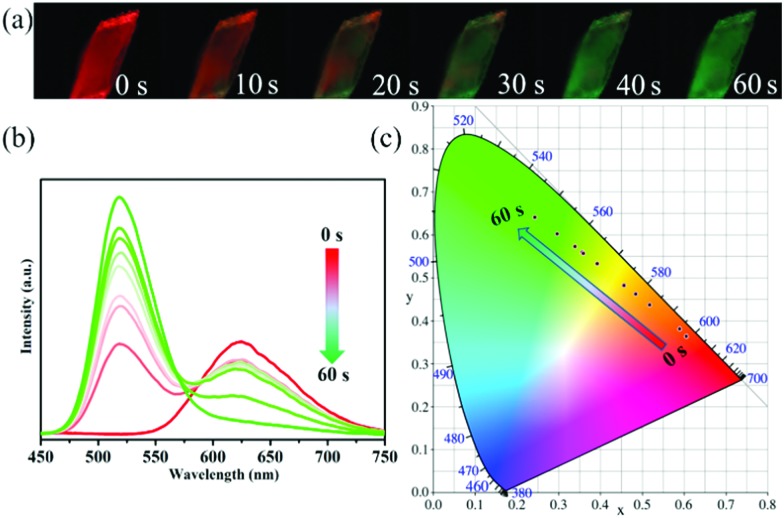
Thermochromic luminescence of compound **1** under thermal treatment at 90 °C. (a and b) Time-dependent PL photos and the emission spectra (*λ*_Ex_ = 360 nm) of crystal **1** at 90 °C. (c) Commission Internationale de l’Eclairage (CIE) chromaticity coordinates corresponding to the emission.

**Fig. 3:**
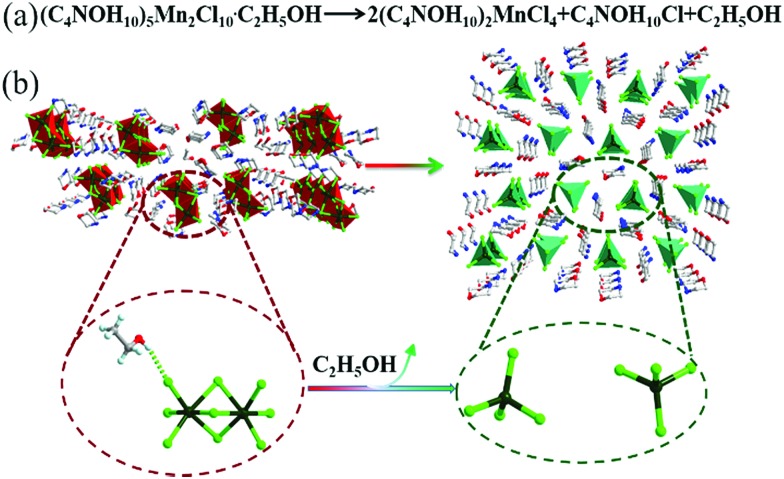
Proposed solvent induced structure conversion reaction. (a) Predicting the balanced reaction of the transition process from compound **1** to **2**. (b) Predicting the transition process from compound **1** to **2**.

To examine the thermally induced structural transformation from compound **1** to **2**, PXRD was used to characterize the structural and phase changes at 90 °C. It is a pity that we could not obtain a high quality single-crystal of **1** after thermal treatment at 90 °C for SCXRD. Nevertheless, we could find evidence in the PXRD pattern of the thermally treated compound **1**, in which diffraction peaks of compound **2** and the C_4_NOH_10_Cl phase appeared and those of compound **1** disappeared, indicating that the compound **1** crystal is transformed into compound **2** (Fig. 4a). In order to further study the structural transformation, thermogravimetric analysis (TGA) and differential scanning calorimetry (DSC) of compound **1** were performed (Fig. 4b). The TGA plot of compound **1** has a small stable plateau at about 90 °C and the material starts to decompose at 150 °C. The decomposition temperature starting point of compound **1** is similar to that of compound **2** and C_4_NOH_10_Cl (ESI Fig. S12†). It is due to the presence of intermolecular hydrogen bonds that compound **1** lost ethanol molecules at 90 °C. In the DSC plot, the change of [Mn_2_Cl_9_]^5–^ is induced by the departure of the ethanol molecule at 90 °C. The PL changes from the red emission of six coordinate [Mn_2_Cl_9_]^5–^ to green emission of four coordinate [MnCl_4_]^2–^ with losing equivalent ethanol, and the whole process is irreversible. As proposed above, a solid state balanced reaction of the transition process from compound **1** to **2** was proposed: (C_4_NOH_10_)_5_Mn_2_Cl_9_·C_2_H_5_OH → 2(C_4_NOH_10_)_2_MnCl_4_ + C_4_NOH_10_Cl + C_2_H_5_OH.

**Fig. 4:**
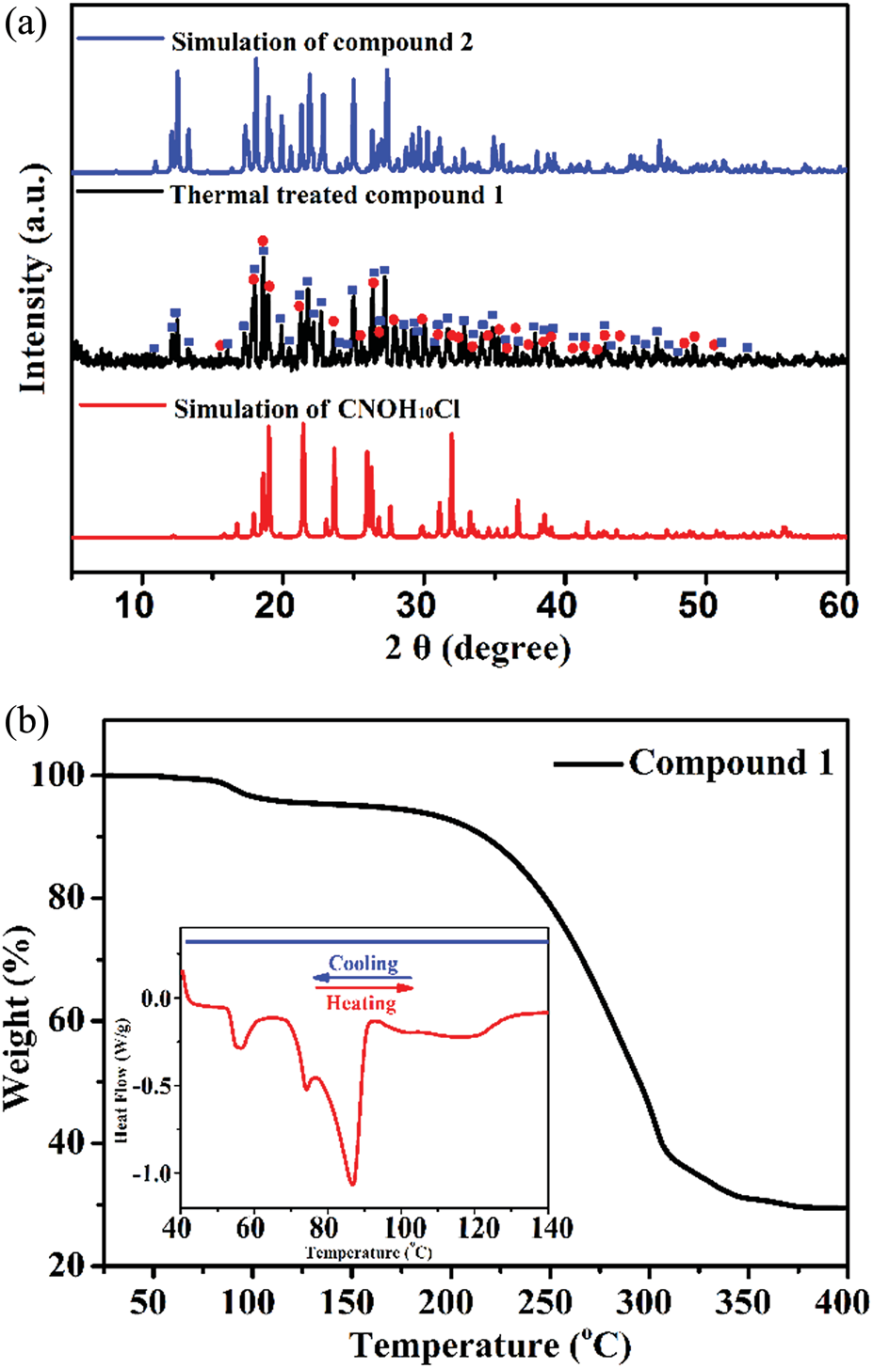
(a) PXRD of the thermally treated **1** crystal (blue squares: patterns from the compound **2** phase; red circles: patterns from the C_4_NOH_10_Cl phase). (b) TGA plot of compound **1** (inset: DSC plot for **1**).

## Conclusions

In summary, we have reported the synthesis and structure of a binuclear organic manganese chloride cluster in octahedral coordination, which demonstrated thermally induced structural transformation into a monomanganese halide in tetrahedral coordination and the synchronous thermochromic luminescence response. The structural transformation involves the cleavage of metal halide bonds followed by structural reorganization, which was characterized by crystallographic analysis, spectroscopic methods and TGA. Our research extends the ability to assemble hybrids of organic–inorganic metal halides with controlled structures in a rational manner and contributes to a better understanding of the thermal stability of metal halides. And the ecofriendly, hypotoxicity, high performance light emitting crystals and rapid conversion of luminescence from red to green represented a major breakthrough in the field of light emitting materials.

## Conflicts of interest

There are no conflicts to declare.

## Supplementary Material

Supplementary information

Crystal structure data
