# Supplementary material for: Thermoinduced structural-transformation and thermochromic luminescence in organic manganese chloride crystals
Source: Chem Sci. 2019 Feb 25;10(13):3836–9. doi: 10.1039/c8sc04711a (PMC6457203; doi:10.1039/c8sc04711a)
Supplement: Supplementary file 1 [file SC-010-C8SC04711A-s001.pdf]

Electronic Supplementary Material (ESI) for Chemical Science.
This journal is © The Royal Society of Chemistry 2019

Supporting Information

Thermoinduced Structural-Transformation and Thermochromic Luminescence in Organic Manganese Chloride Crystals

Meng-En Sun, [a] Yao Li, [a] Xi-Yan Dong, [a, b] Shuang-Quan Zang [a, *]

[a]College of Chemistry and Molecular Engineering, Zhengzhou University, Zhengzhou 450001, China.

E-mail: zangsqzg@zzu.edu.cn

[b]College of Chemistry and Chemical Engineering, Henan Polytechnic University, Jiaozuo 454000, China

Materials and Methods

Materials. All reagents and solvents used were of commercially available reagent grade and were used without any additional purification.

Characterization

Thermogravimetric analysis (TGA) of the compounds were performed on a SDT 2960 thermal analyzer from room temperature to 400℃ at a heating rate of 5℃/min under nitrogen atmosphere. UV-vis absorption spectra was recorded with a U-2000 spectrophotometer. Powder X-ray diffraction (PXRD) data were collected at room temperature in air using X'Pert PRO diffractometer (Cu Kα, λ = 1.54178 Å). For better peaking, the PXRD data for the heat treated samples was de-baseline treated with X'Pert HighScore Plus. Simulated powder patterns were calculated by Mercury software using the crystallographic information file (CIF) from single-crystal x-ray experiment.

Differential Scanning Calorimeter (DSC). DSC analyses of the compound were performed on a DSC Q25 thermal analyzer from room temperature to150℃ and cool to room temperature at a heating rate of 5℃/min under nitrogen atmosphere.

Single-Crystal X-ray Diffraction Analysis (SCXRD). SCXRD measurements were performed on a Rigaku XtaLAB Pro diffractometer with Mo-Kα radiation (λ = 0.7107 Å) at 293K for Data collection and reduction were performed using the program CrysAlisPro. The intensities were corrected for absorption using empirical method implemented in SCALE3 ABSPACK scaling algorithm. The crystal structures are visualized by DIAMOND 3.2.

Luminescence measurements. Luminescence microscopy images were recorded on an Olympus BX53 microscope, and measurements were carried out using a HORIBA FluoroLog-3 fluorescence spectrometer. Luminescence decay were measured on HORIBA Scientific Fluorolog-3 spectrofluorometer equipped with phosphor lamp, operating in time-correlated single photon counting mode (TCSPC) with a resolution time of 200 ps. The photoluminescent quantum efficiency in powder form was measured using an integrating sphere on a HORIBA Scientific Fluorolog-3 spectrofluorometer.

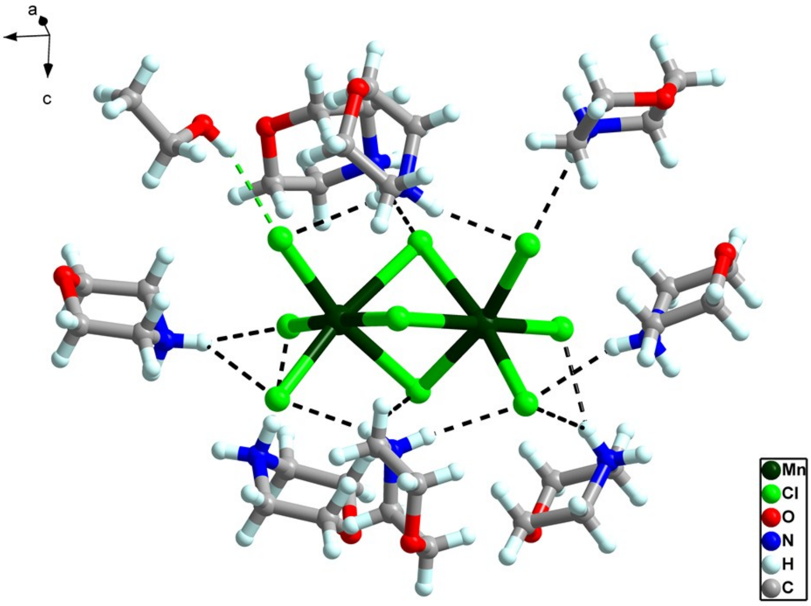

Figure S1. View of an individual $[Mn_2Cl_9]^{5-}$ anion and nearest neighbor $C_4NOH_{10}{}^{+}$ cations in compound $(C_4NOH_{10})_5Mn_2Cl_9{\cdot}C_2H_5OH$ (**1**). N–H···Cl hydrogen-bonding interactions between $C_4NOH_{10}{}^{+}$ and $[Mn_2Cl_9]^{5-}$(black dash line) the N–H···Cl (N–H···Cl angles) are 3.205 Å (147.92°), 3.198 Å (171.57°), 3.340 Å (138.89°), 3.337 Å (134.29°), 3.556 Å (128.49°), 3.287 Å (147.64°), 3.218 Å (163.40°), 3.410 Å (169.02°), 3.359 Å (111.03°), 3.217 Å (158.78°),3.335 Å (172.82°), 3.155 Å (154.96°) and 3.278 Å (154.27°), respectively. O–H···Cl hydrogen-bonding interactions between $[Mn_2Cl_9]^{5-}$ and C_2H_5OH (green dash line); the O–H···Cl (O–H···Cl angles) are 3.226 Å (155.42°).

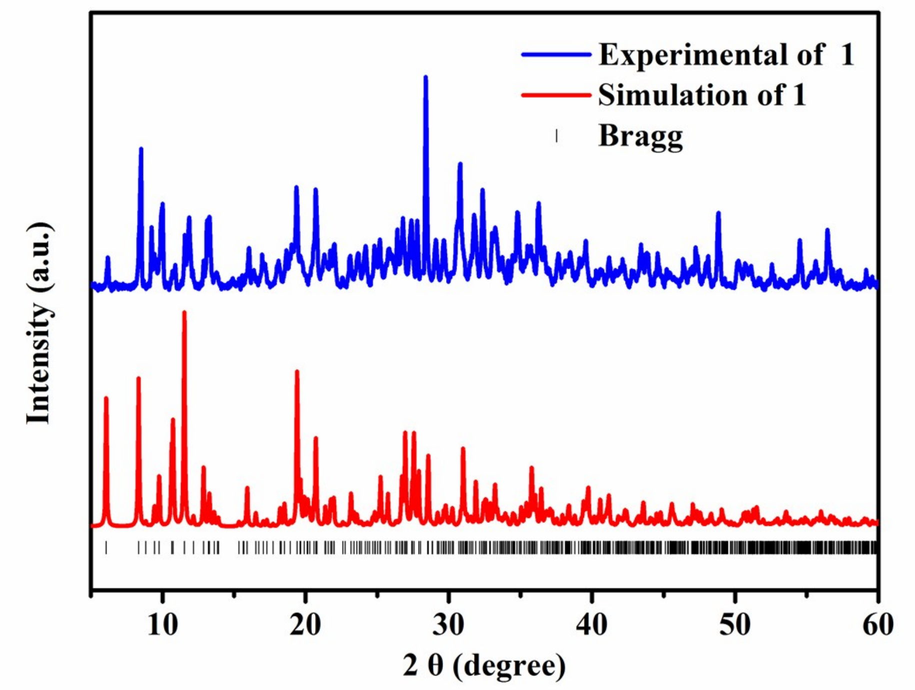

Figure S2. X-Ray powder diffraction (PXRD) patterns of compound **1** crystals powder (blue line) and simulated from single-crystal data (red line).

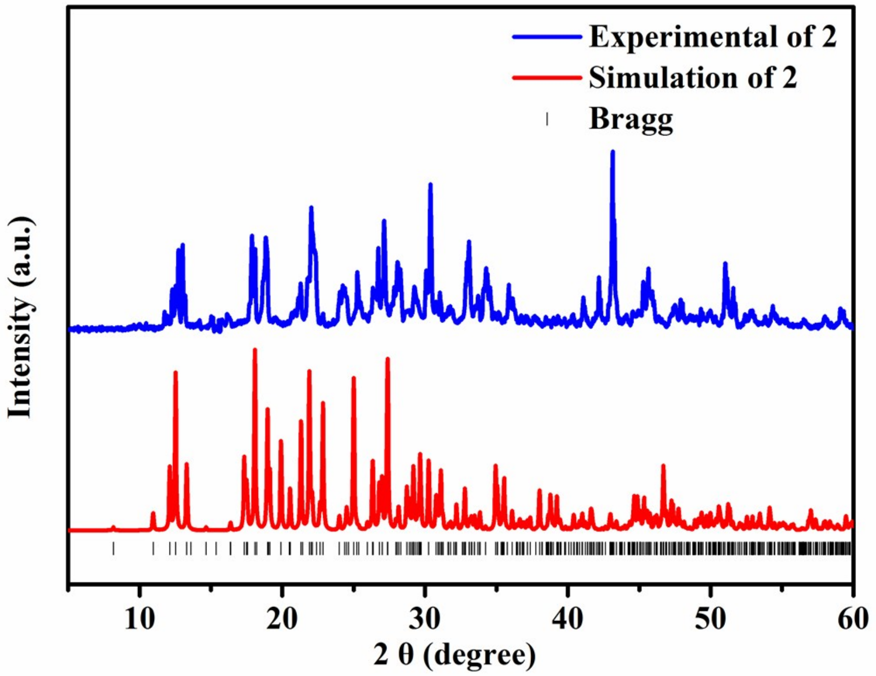

Figure S3. X-Ray powder diffraction (PXRD) patterns of compound **2** crystals powder (blue line) and simulated from single-crystal data (red line).

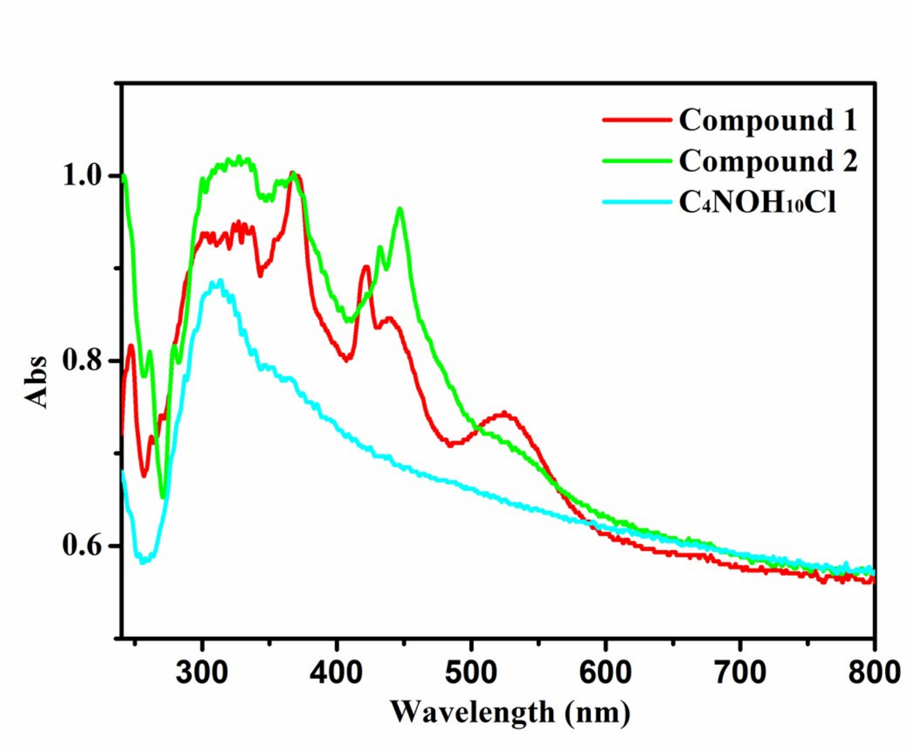

Figure S4. UV-vis diffuse reflectance spectra of solid-state compounds **1** (red line), **2** (green line) and $C_4NOH_{10}Cl$ (turquoise line).

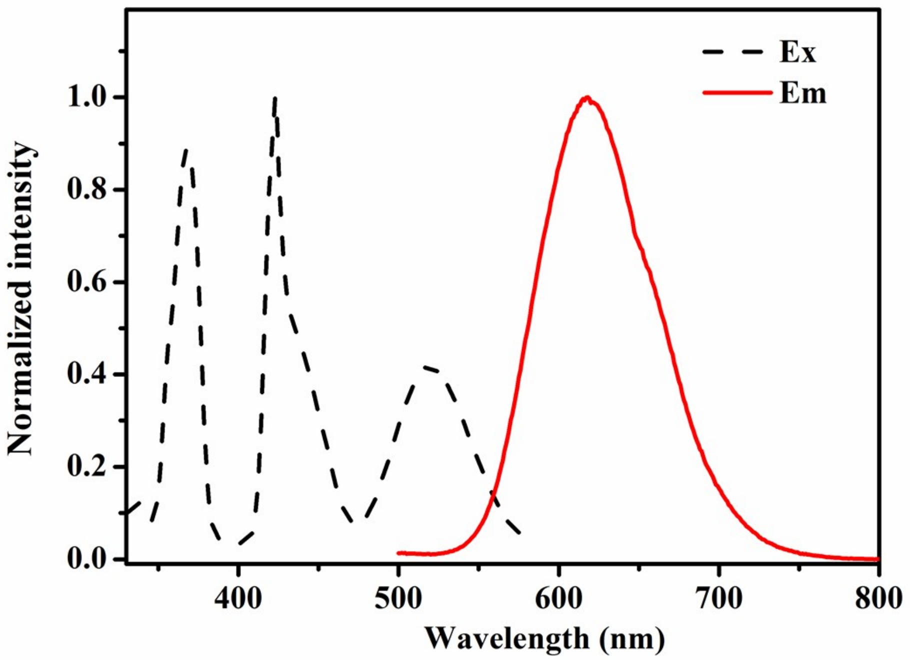

Figure S5. Excitation spectra (monitored at 620 nm) (dotted line) and emission spectra (excited at 420 nm) (solid line) of solid-state compound **1** at R.T.. Em = Emission, Ex = Excitation. The intensity of peaks was normalized.

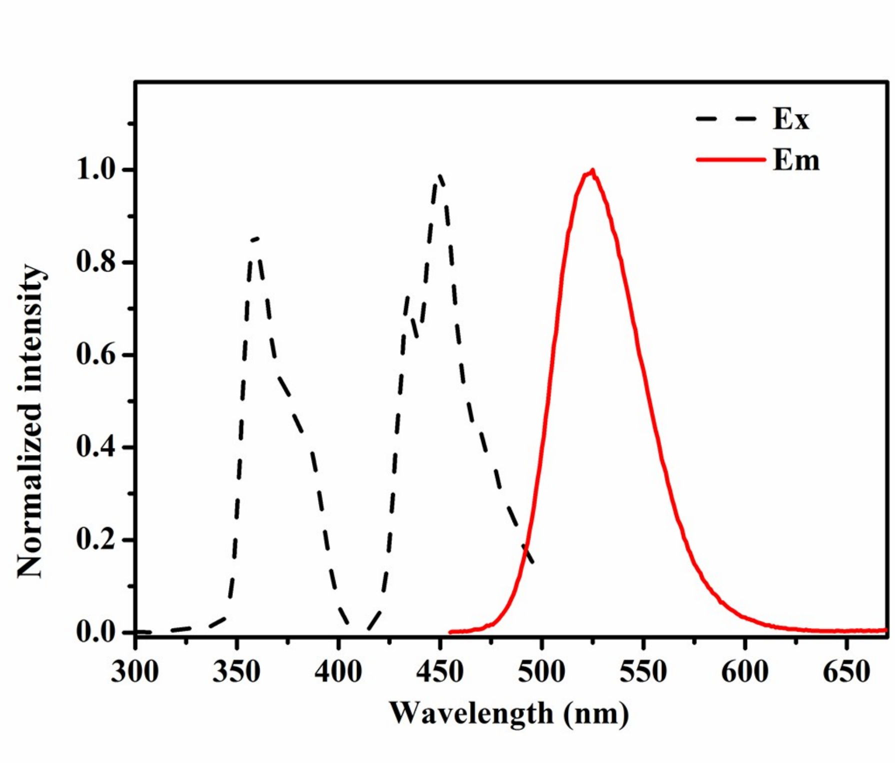

Figure S6. Excitation spectra (monitored at 520 nm) (dotted line) and emission spectra (excited at 430 nm) (solid line) of solid-state compound **2** at R.T.. Em = Emission, Ex = Excitation. The intensity of peaks was normalized.

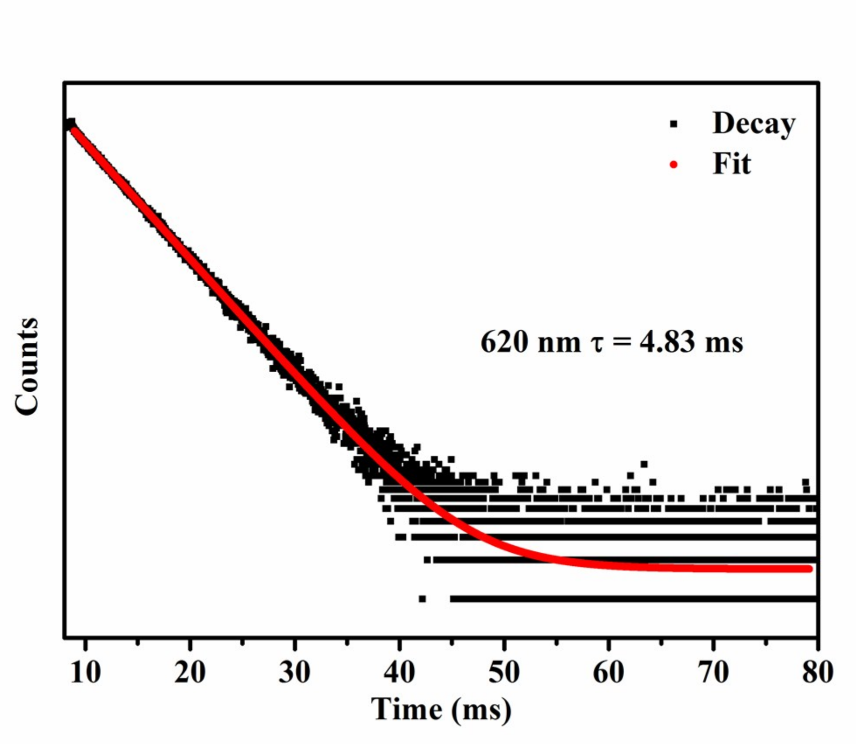

Figure S7. Representative time-resolved photoluminescence decays (λ_{Ex} = 420 nm) of solid-state compound **1** at R.T..

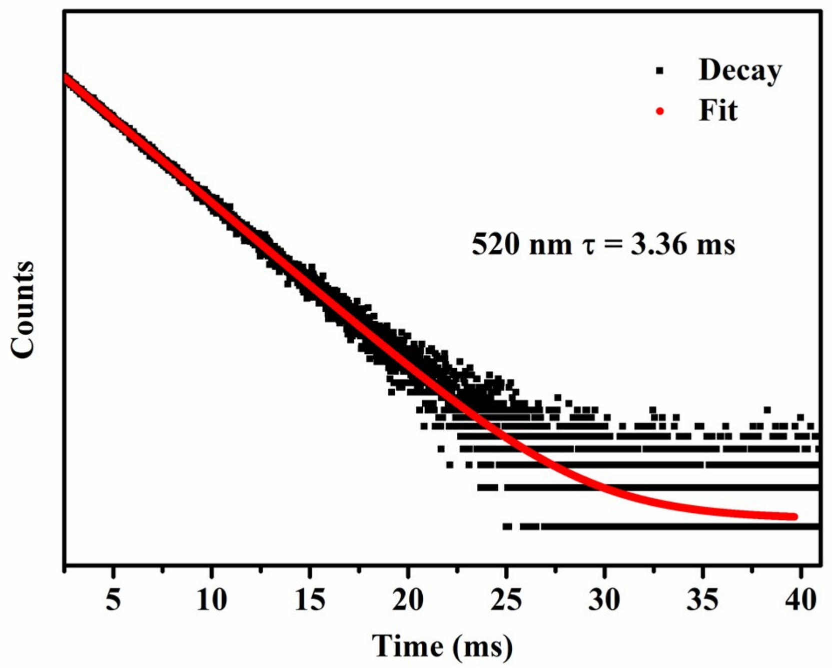

Figure S8. Representative time-resolved photoluminescence decays (λ_{Ex} = 430 nm) of solid-state compound **2** at R.T..

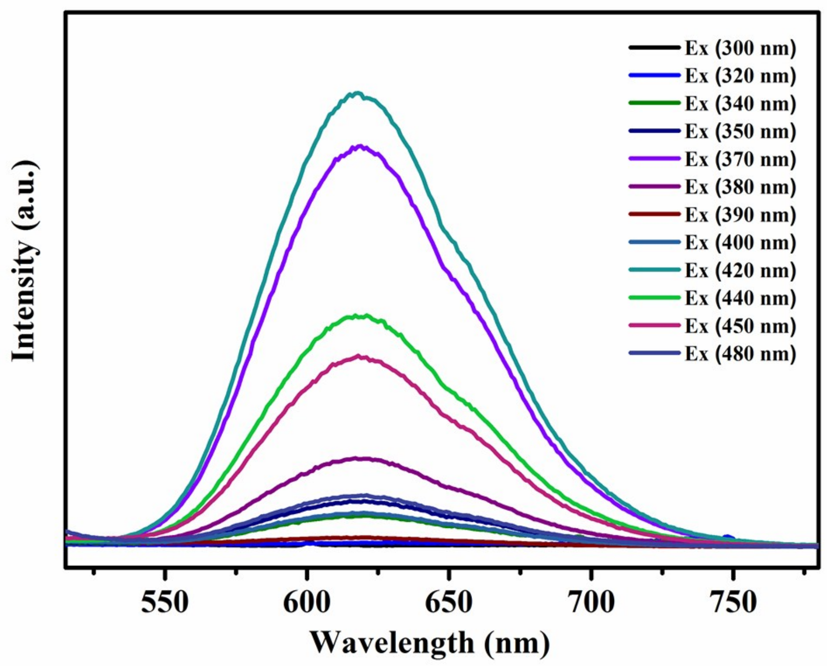

Figure S9. The excited wavelength-dependent emission spectra of solid-state compound **1** at R.T..

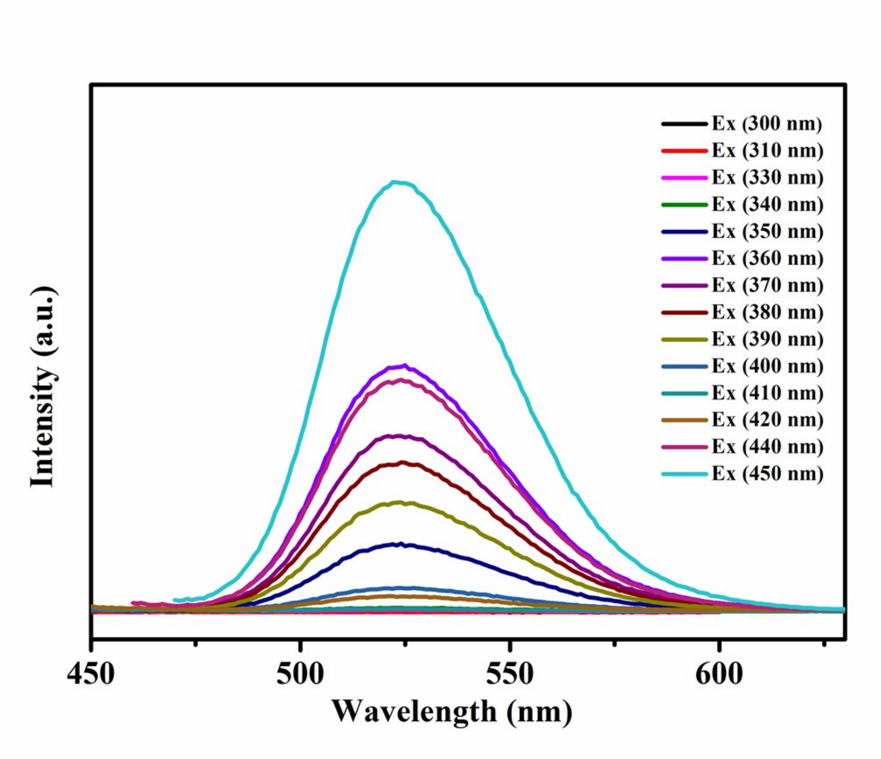

Figure S10. The excited wavelength-dependent emission spectra of solid-state compound **2** at R.T..

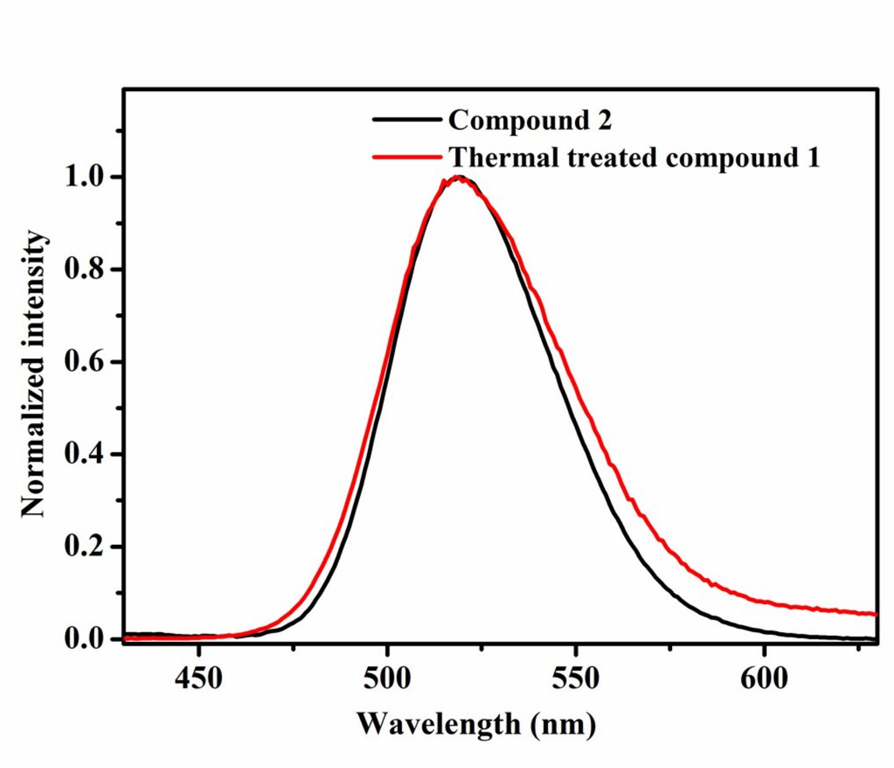

Figure S11. The luminescence spectra of solid-state compound **2** (black line, λ_{Ex} = 360 nm) and thermal treated compound **1** (red line, λ_{Ex} = 360 nm). The intensity of peaks was normalized.

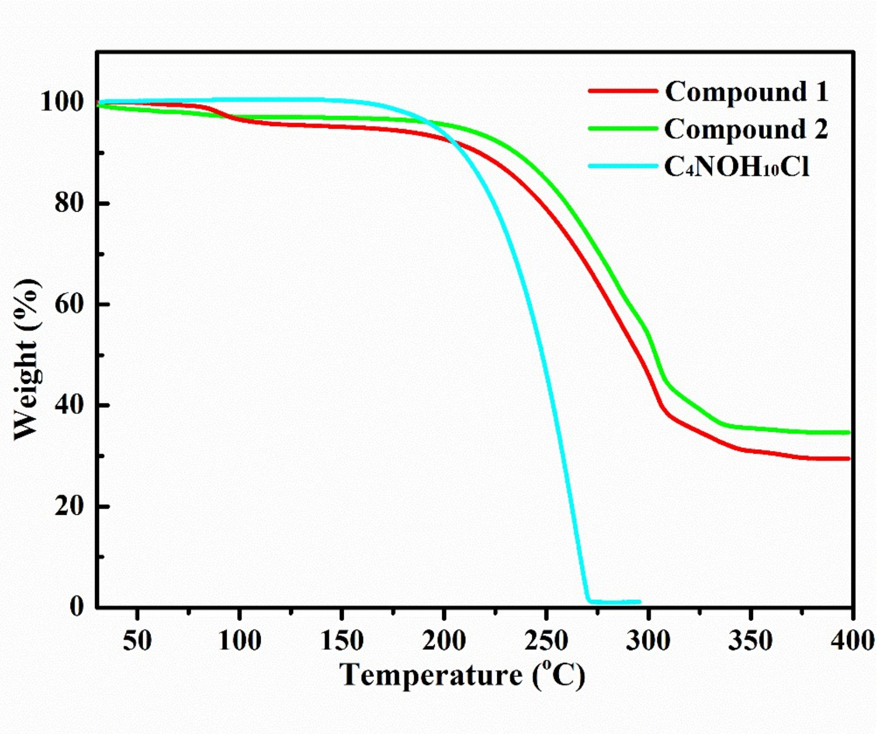

Figure S12. TGA plot of compounds **1** (red line), **2** (green line) and $C_4NOH_{10}Cl$ (turquoise line).

Table S1. Single crystal X-ray diffraction data of compounds **1** and **2** crystals.

Compound	$(C_4NOH_{10})_5Mn_2Cl_{10}$·EtOH	$(C_4NOH_{10})_2MnCl_4$
Formula weight	915.64	373.00
Temperature/K	100.01(10)	149.99(10)
Crystal system	orthorhombic	monoclinic
Space group	$P2_12_12_1$	$P2_1/c$
a/Å	9.0363(6)	6.5440(2)
b/Å	20.0218(13)	16.1548(4)
c/Å	21.2177(15)	14.6769(4)
α/°	90	90
β/°	90	95.619(3)
γ/°	90	90
Volume/Å^3	3838.8(4)	1544.14(7)
Z	4	4
ρ_{calc} g/cm^3	1.584	1.604
μ/mm^{-1}	1.326	1.539
F(000)	1896.0	764.0
Radiation(Mo Kα)	0.71073	0.71073
2θ range for data collection/°	4.946 to 58.396	3.76 to 57.894
Reflections collected	16494	12384
Data/restraints/parameters	8196/0/399	3588/0/154
Goodness-of-fit on F^2	1.028	1.045
Final R indexes [I>=2σ (I)]	R_1= 0.0407, wR_2= 0.0849	R_1= 0.0225, wR_2= 0.0570
Final R indexes [all data]	R_1= 0.0522, wR_2= 0.0873	R_1= 0.0254, wR_2= 0.0582
Flack parameter	0.03(2)	
CCDC number.	1867428	1867436

$R_1 = \sum||F_o|-|Fc|\sum/||F_o|$. $wR_2 = [\sum w(F_o^2 - F_c^2)^2/\sum w(F_o^2)^2]$

Table S2. Atomic positions of compound **1**. All non-hydrogens were refined with anisotropic displacement parameters, while the hydrogens were refined with isotropic displacement parameters.

Atom	x	y	z	U(eq)
Mn1	1756.0(8)	4200.3(4)	6848.2(4)	10.45(17)
Mn2	1501.7(8)	2489.6(4)	6924.7(4)	10.61(17)
Cl1	2122.0(14)	4851.1(6)	7849.2(6)	13.8(3)
Cl2	-203.3(13)	4986.5(6)	6508.0(6)	13.2(3)
Cl3	3845.0(14)	4734.7(6)	6247.3(6)	14.8(3)
Cl4	-161.5(13)	3401.3(6)	7373.0(5)	12.0(2)

Cl5	3575.2(13)	3309.7(6)	7226.7(5)	10.2(2)
Cl6	1465.4(13)	3299.5(6)	5896.5(5)	12.3(2)
Cl7	1800.7(14)	1910.5(6)	7980.0(6)	14.3(3)
Cl8	-567.7(14)	1770.1(6)	6617.2(6)	15.2(3)
Cl9	3566.4(13)	1799.0(5)	6407.6(6)	12.4(2)
O1	2969(4)	3087.3(19)	9837.2(17)	21.4(9)
O2	-3366(4)	4449.7(18)	8428.5(18)	24.0(9)
O3	2253(4)	5121.0(18)	4564.3(18)	19.5(9)
O4	7624(4)	3511.0(19)	5219.1(17)	19.4(9)
O5	-3629(4)	2321.2(18)	8460.0(18)	22.6(9)
O6	-4518(5)	5563(2)	5145.5(19)	29.6(10)
N1	2269(4)	3441(2)	8570.3(19)	11.6(9)
N2	-1877(5)	5461(2)	7734(2)	18.8(10)
N3	267(5)	4035(2)	4660(2)	14.0(9)
N4	5064(5)	3160(2)	5917(2)	13.7(9)
N5	-1864(5)	1261(2)	7983(2)	15.9(10)
C1	1485(6)	3040(3)	9613(3)	21.4(12)
C2	1187(6)	3531(2)	9090(2)	14.6(11)
C3	3816(5)	3458(3)	8814(2)	13.3(11)
C4	3998(6)	2955(3)	9338(2)	19.0(12)
C5	-3680(6)	4544(3)	7777(3)	25.6(14)
C6	-3441(6)	5258(3)	7590(3)	20.7(12)
C7	-1490(6)	5302(3)	8402(2)	17.5(11)
C8	-1829(6)	4584(3)	8541(3)	19.5(12)
C9	1074(6)	5132(3)	5016(2)	17.0(11)
C10	-229(6)	4734(3)	4782(3)	15.1(11)
C11	1571(6)	4016(3)	4224(2)	17.1(11)
C12	2769(6)	4451(3)	4481(3)	18.3(12)
C13	6199(6)	3546(3)	4933(3)	19.6(12)
C14	5159(6)	3041(3)	5226(2)	18.6(12)
C15	6561(6)	3168(2)	6216(2)	15.9(11)
C16	7520(6)	3666(3)	5875(2)	16.6(11)
C17	-2128(6)	2247(3)	8646(3)	19.6(12)
C18	-1662(6)	1519(2)	8634(2)	18.8(12)
C19	-3409(6)	1370(3)	7758(3)	20.1(12)
C20	-3816(7)	2100(3)	7823(3)	23.4(13)
C21	-3473(7)	5819(3)	5581(3)	22.5(12)
C22	-2369(6)	6228(3)	5220(3)	23.0(13)

Table S3. Atomic positions of compound **2**. All non-hydrogens were refined with anisotropic displacement parameters, while the hydrogens were refined with isotropic displacement parameters.

Atom	x	y	z	U(eq)
Mn1	-4397.5(3)	6625.5(2)	2758.6(2)	18.08(7)
Cl1	-5535.5(6)	6413.6(2)	1200.3(2)	28.64(9)
Cl2	-707.1(5)	6713.3(2)	2852.8(2)	21.94(8)
Cl3	-5205.1(5)	5468.0(2)	3648.9(2)	20.79(8)
Cl4	-5451.0(5)	7819.8(2)	3491.3(3)	28.33(9)
O1	-333.9(14)	4185.2(6)	657.7(6)	19.4(2)
O2	450.5(14)	3946.5(6)	4133.2(7)	24.3(2)
N1	599.8(17)	4763.1(7)	2487.6(8)	19.7(2)
N2	-1012.5(18)	2579.0(7)	5097.4(8)	22.9(3)
C1	1715(2)	4424.8(9)	989.0(9)	20.2(3)
C2	2108(2)	4276.7(9)	2001.5(9)	21.3(3)
C3	-1544(2)	4555.8(9)	2110.2(10)	22.5(3)
C4	-1772(2)	4682.1(9)	1088.5(10)	22.0(3)
C5	1834(2)	3309.3(9)	4474.8(11)	26.6(3)
C6	748(2)	2491.7(9)	4526.1(11)	26.4(3)
C7	-2390(2)	3270.0(9)	4749.7(11)	23.6(3)
C8	-1151(2)	4050.2(9)	4724.0(10)	23.5(3)

Table S4. Selected bond lengths (Å) and bond angles (°) for compounds **1** and **2**.

$(C_4NOH_{10})_5Mn_2Cl_{10}·EtOH$ (**1**)				$(C_4NOH_{10})_2MnCl_4$ (**2**)			
**Bond lengths (Å)**		**Bond Angles (°)**		**Bond lengths (Å)**		**Bond Angles (°)**	
**Mn1-Cl1**	**2.5137(14)**	**Cl1-Mn1-Cl3**	**96.25(5)**	**Mn1-Cl1**	**2.3600(4)**	**Cl1-Mn1-Cl2**	**106.469(15)**
**Mn1-Cl2**	**2.4765(14)**	**Cl1-Mn1-Cl4**	**92.56(5)**	**Mn1-Cl2**	**2.4096(4)**	**Cl1-Mn1-Cl3**	**110.705(15)**
**Mn1-Cl3**	**2.5167(14)**	**Cl1-Mn1-Cl5**	**90.66(4)**	**Mn1-Cl3**	**2.3703(4)**	**Cl3-Mn1-Cl2**	**106.926(14)**
**Mn1-Cl4**	**2.6080(14)**	**Cl1-Mn1-Cl6**	**169.63(5)**	**Mn1-Cl4**	**2.3450(4)**	**Cl4-Mn1-Cl1**	**118.994(16)**
**Mn1-Cl5**	**2.5548(13)**	**Cl2-Mn1-Cl1**	**90.62(5)**			**Cl4-Mn1-Cl2**	**105.264(14)**
**Mn1-Cl6**	**2.7201(14)**	**Cl2-Mn1-Cl3**	**96.80(5)**			**Cl4-Mn1-Cl3**	**107.750(15)**
**Mn2-Cl4**	**2.5487(14)**	**Cl2-Mn1-Cl4**	**92.26(5)**				
**Mn2-Cl5**	**2.5725(14)**	**Cl2-Mn1-Cl5**	**174.41(5)**				
**Mn2-Cl6**	**2.7184(13)**	**Cl2-Mn1-Cl6**	**97.82(4)**				
**Mn2-Cl7**	**2.5360(14)**	**Cl3-Mn1-Cl4**	**167.28(5)**				
**Mn2-Cl8**	**2.4489(14)**	**Cl3-Mn1-Cl5**	**88.47(5)**				
**Mn2-Cl9**	**2.5681(13)**	**Cl3-Mn1-Cl6**	**88.75(4)**				
		**Cl4-Mn1-Cl6**	**81.15(4)**				
		**Cl5-Mn1-Cl4**	**82.25(4)**				
		**Cl5-Mn1-Cl6**	**80.37(4)**				
		**Cl4-Mn2-Cl5**	**83.07(4)**				
		**Cl4-Mn2-Cl6**	**82.25(4)**				
		**Cl4-Mn2-Cl9**	**166.75(5)**				
		**Cl5-Mn2-Cl6**	**80.09(4)**				
		**Cl7-Mn2-Cl4**	**93.48(5)**				
		**Cl7-Mn2-Cl5**	**89.67(4)**				
		**Cl7-Mn2-Cl6**	**169.29(5)**				
		**Cl7-Mn2-Cl9**	**93.08(4)**				
		**Cl8-Mn2-Cl4**	**94.04(5)**				
		**Cl8-Mn2-Cl5**	**176.35(5)**				
		**Cl8-Mn2-Cl6**	**97.35(5)**				
		**Cl8-Mn2-Cl7**	**92.73(5)**				
		**Cl8-Mn2-Cl9**	**97.14(5)**				
		**Cl9-Mn2-Cl5**	**85.47(4)**				
		**Cl9-Mn2-Cl6**	**89.26(4)**				
		**Mn1-Cl5-Mn2**	**84.18(4)**				
		**Mn2-Cl4-Mn1**	**83.58(4)**				
		**Mn2-Cl6-Mn1**	**84.18(4)**				

Table S5. The excited wavelength-dependent emission decay time for compounds **1** and **2**.

$(C_4NOH_{10})_5Mn_2Cl_{10}·EtOH$ (**1**)		$(C_4NOH_{10})_2MnCl_4$ (**2**)	
Excitation wavelength (nm)	Lifetime (ms)	Excitation wavelength (nm)	Lifetime (ms)
360	4.74	350	3.34
380	4.61	380	3.36
420	4.84	400	3.32
440	4.82	430	3.36
500	4.78	470	3.35
530	4.83	490	3.31
